# Temperature-Controlled Solvent Vapor Annealing of Thin Block Copolymer Films

**DOI:** 10.3390/polym11081312

**Published:** 2019-08-06

**Authors:** Xiao Cheng, Alexander Böker, Larisa Tsarkova

**Affiliations:** 1 Fraunhofer Institute for Applied Polymer Research IAP, Geiselbergstr. 69, 14476 Potsdam-Golm, Germany; 2 Lehrstuhl für Polymermaterialien und Polymertechnologie, University of Potsdam, 14476 Potsdam-Golm, Germany; 3 Deutsches Textilforschungszentrum Nord-West (DNTW), Adlerstr. 1, 47798 Krefeld, Germany; 4 Chair of Colloid Chemistry, Department of Chemistry, Moscow State University, Leninskie Gory 1-3, 119991 Moscow, Russia

**Keywords:** solvent vapor annealing, block copolymer films, ellipsometry, guided self assembly

## Abstract

Solvent vapor annealing is as an effective and versatile alternative to thermal annealing to equilibrate and control the assembly of polymer chains in thin films. Here, we present scientific and practical aspects of the solvent vapor annealing method, including the discussion of such factors as non-equilibrium conformational states and chain dynamics in thin films in the presence of solvent. Homopolymer and block copolymer films have been used in model studies to evaluate the robustness and the reproducibility of the solvent vapor processing, as well as to assess polymer-solvent interactions under confinement. Advantages of utilizing a well-controlled solvent vapor environment, including practically interesting regimes of weakly saturated vapor leading to poorly swollen states, are discussed. Special focus is given to dual temperature control over the set-up instrumentation and to the potential of solvo-thermal annealing. The evaluated insights into annealing dynamics derived from the studies on block copolymer films can be applied to improve the processing of thin films of crystalline and conjugated polymers as well as polymer composite in confined geometries.

## 1. Introduction

Polymer films represent one of the most used classes of soft matter with applications ranging from functional coatings and sensors to separation membranes and organic electronics. Along with the chemical composition, the conformations of macromolecular chains determine the properties of polymer films such as glass transition temperature (*T*_g_) [1,2], electron densities [3], wetting [4,5], rheology [6], solvent absorption, and swelling dynamics [7,8]. Extensive research has revealed that characteristics of thin polymer films strongly deviate from the properties these polymers exhibit in bulk [9]. In particular, as-cast films have been reported to have a substantially reduced effective viscosity as compared to annealed films [6,10]. The effect was presumably attributed to the reduced entanglement density caused by the rapid quenching of non-equilibrium chain conformations during spin coating. Despite partial progress in the understanding, it is becoming increasingly obvious that a clear comprehension of thin polymer film behavior has not been yet achieved [9,11,12]. Advancing the understanding and the usage of polymer-based materials requires that film preparation and film processing reliably and reproducibly bring the system to a desired (quasi) equilibrium state [2,13]. This requirement is especially useful for annealing of high-molecular weight and high χ-parameter block copolymers. 

To relax the kinetic barriers formed by non-equilibrium states and residual stresses in polymer films, they are routinely subjected to thermal annealing at temperatures well above *T*_g_. However, certain polymers with complex compositions possess a narrow temperature gap between *T*_g_ and the decomposition temperature, thus equilibrium conformations are not achievable within acceptable annealing times. Also, the segmental dynamics of polymer chains in thin films are very much retarded by the multiple chain contacts with the solid surface, and therefore polymer dynamics in thin films are only moderately accelerated with increasing temperature [14]. Therefore, processing of polymer films under solvent vapor atmosphere, i.e., solvent vapor annealing, is considered to be a promising alternative to the methods currently prevailing in technological fabrication thermal annealing.

A challenging research problem concerns the influence of confinement on the solvent absorption and on the related chain mobility in swollen films [15]. A possible reason for qualitatively differing results is the phenomenological complexity of diffusion and equilibrium uptake of small molecules by the confined polymer matrix. Along with the substrate effects, the conformational states of polymer chains imposed by confined geometry and film preparation affect the physical properties and the behavior of thin polymer films. 

In this respect, intensive efforts are currently devoted to the issue of controlled swelling and guidance of block copolymer self-assembly in confined geometries towards targeted structures and functions [16,17,18,19,20,21,22,23,24,25,26,27]. The research on thin block copolymer films has significantly advanced the understanding of the mechanisms behind the solvent vapor annealing owing to the possibility to visualize the development of microphase-separated structures during processing. The gained knowledge governed the design of instrumental set-ups, which reproducibly provide time- and process-effective annealing [21,28,29,30,31,32,33,34,35,36,37,38]. Exposure of block copolymer films to solvent vapors serves multiple purposes, including establishing surface/interface preferentiality towards particular blocks [39,40,41,42,43,44,45,46], increasing polymer chain mobility by decreasing effective *T*_g_ [47], as well as reducing interactions of the polymer chains with the substrate [48,49,50]. Also, addition of solvents typically induces changes of domain spacings [18,51,52,53,54,55,56] or of the morphological behavior by altering χ_eff_ parameters [41,57,58,59,60] and relative volume fractions of the blocks in the case of selective solvents [18,26,61,62,63,64]. The concentration of the solvent in the film defines the swollen thickness and thus can also affect the microdomain formation [25,33,65,66,67,68,69,70]. Solvent vapor annealing is now routinely used to guide the structure formation into targeted patterns [21,25,26,27]. Moreover, high sensitivity of the microphase separation to the concentration of the solvent in a swollen film allows for the evaluation of the effect of the nano-structuring of polymer films on the solvent uptake [46,71,72,73] as well as the assessment of the reproducibility of the annealing conditions [66].

Instrumentation of solvent vapor annealing set-up has developed from a simple sealed container with a vial with solvent inside (static annealing) [16,32], a chamber with additional input and output tubes to control the deswelling rate [74], to a dynamic annealing channel system with external vessels and an automated flow of carrier gas (nitrogen) to produce a controlled solvent vapor atmosphere [28,29,41]. Recently, the systems with a feedback loop, which can automatically maintain a constant degree of swelling, were reported by Jin et al. [19]. The annealing system with pneumatic valves that can precisely control the annealing time in millisecond range was designed by Nelson et al. [28]. These studies delivered significant input into developing the solvent vapor annealing procedure up to reproducibility levels required for industrial technological processing. Several papers have reported applications other than ambient solvent vapor annealing [30,37,75,76,77,78,79]. In particular, warm solvent vapor annealing of polystyrene-*b*-polyvinyl pyridine block copolymer at solvent temperatures as high as 60 °C has been shown to reduce the annealing time from hours to several minutes [75]. The importance of the temperature control, particularly the possibility to adjust the temperatures of the substrate and of the vapor independently, is recognized in the field of thin functional films, and systematic qualitative studies on this issue may expand the applications of these methods [20,27,80,81,82].

In this paper, we assess experimental factors that affect the reproducibility and the stability of the solvent vapor atmosphere during annealing. All annealing experiments are performed with in-situ measurements of the swollen film thickness, which is the most important information to verify the stability of the processing conditions. The paper is organized as follows. We first introduce the technical features of the annealing system such as design, type of volumetric flow, and material of the annealing chamber. Then, temperature-controlled swelling of polymer films (solvo-thermal annealing) is introduced on a quantitative basis. Finally, the swelling behaviors of homopolymers polystyrene (PS), poly (2-vinyl pyridine) (P2VP), and the block copolymer polystyrene-*b*-poly(2-vinyl pyridine) (PS-PVP) are analyzed to evaluate the solvent selectivity to the block copolymer components. 

## 2. Materials and Methods

### 2.1. Materials

Polystyrene-*b*-poly(2-vinyl pyridine) (denoted here as PS-PVP) diblock copolymer with a total molecular weight of *M*_n_ = 390 kg/mol and a volume fraction of polystyrene block of 0.48 was synthesized by sequential living anionic polymerization [83]. Molecular weight and volume composition were characterized by gel permeation chromatography (GPC) and nuclear magnetic resonance (NMR), respectively. The degree of polymerization ~3609 and the interaction parameter χ_PS-P2VP_ ~ 0.178 at room temperature [84] resulted in a high segregation strength of χN ≈ 640. A characteristic spacing in bulk of ~117 nm was assessed by small angle X-ray scattering (SAXS) (at European Synchrotron Radiation Facility, Grenoble, France) by measuring solvent-cast µ-thick PS-PVP films. PS with a molecular weight of *M*_n_ = 184 kg/mol was purchased from PSS Polymer Standards Service GmbH and used as received. P2VP with a molecular weight of *M*_n_ = 105 kg/mol was synthesized by anionic polymerization. Toluene and chloroform were both purchased from Sigma-Aldrich Corporation (St. Louis, MO, USA) and used without further purification. P-type Si wafers (Crys Tec GmbH, Berlin, Germany) with ~2 nm thick SiO_x_ layers were cut in ca 1 × 1 cm^2^ pieces and stored in toluene. Before usage, silicon substrates were additionally cleaned by CO_2_ snow-jet gun and then treated with air plasma at 60 W for 1 min.

### 2.2. Preparation of the Films

Solutions of PS-PVP in toluene were filtered through a Teflon (PTFE) membrane with a pore diameter of 200 nm. Toluene is a selective solvent toward P2VP block, which results in a micellar morphology of the block copolymer in solution and in as-spin-coated films. Then, 40 nm thick PS-PVP films were prepared by spin-coating of 1 wt % polymer solution at a rotational velocity of 2500 rpm on freshly cleaned silicon substrates. Experiments on the swelling of films with varied film thickness are beyond the scope of this study, since they require a proper evaluation of the effect of the residual solvent [85,86].

### 2.3. Characterization of the Films

Swelling behavior of polymer films was monitored by in situ spectroscopic ellipsometry (Omt Imaging, mm30 series, Ulm, Germany). Optical data were collected within a spectral range of 450–800 nm at an incidence angle of 70° using software VisuEl 3.8 from Omt. Film thicknesses were evaluated using Cauchy model and Scout Software (Omt, Ulm, Germany). Scanning force microscopy (SFM) was done using Icon Dimension (Bruker, Billerica, MA, USA) in TappingMode® using tips from OTESPA with spring constant k = 42 N/m. The images were analyzed using software Nanoscope Analysis 1.50 (Bruker). Optical microscopy studies were performed using Axioplan 2 Imaging microscope from Zeiss (Oberkochen, Germany).

### 2.4. Description of the Annealing Set-up

**Figure 1:**
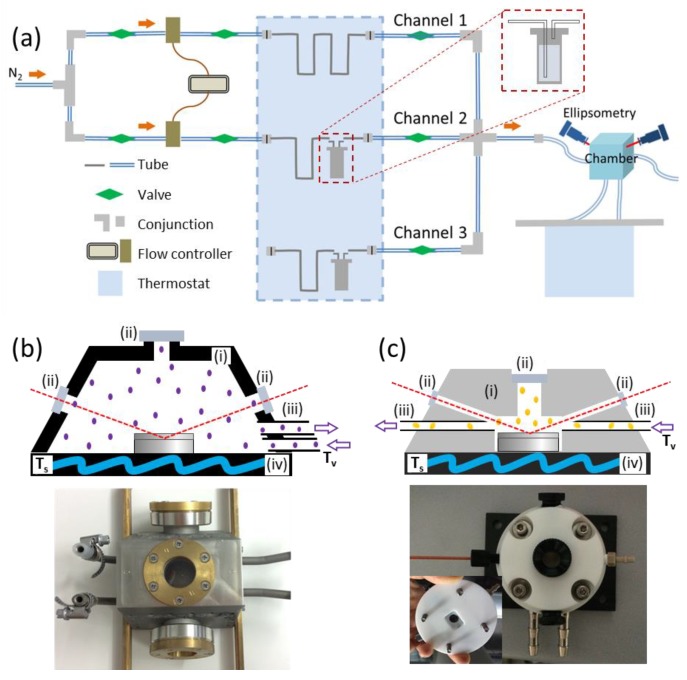
(**a**) The sketch of the annealing system; the tubing and the vessels with solvent in the blue-marked area immersed in the thermostated bath [66]. (**b**,**c**) Sketches and photos of the two annealing chambers made from (**b**) steel and (**c**) Teflon (PTFE). Both chambers contain (i) transparent top windows for the macroscopic observations of the film surface and side windows (not shown in the sketches) for in situ monitoring of the thickness, (ii) inlet and outlet connections for the tubing, and (iii) water-filled tubing in the bottom for adjusting the temperature of the substrate.

Figure 1a presents the designation of the components of the annealing system. Nitrogen, as the carrier gas, passes through two flow controllers MKS 647C (MKS Instruments GmbH, Munich, Germany) connected to the channel with a flow of pure nitrogen (Channel 1) and to the channel that delivers solvent vapor (Channel 2) into the chamber. Flow controllers define the total flow through a channel (a maximum value of 100 sccm), as well as the partial vapor pressure in the chamber p/p_0_. The latter is adjusted by mixing the flows through Channel 1 and Channel 2. We note that the values of p/p_0_ evaluated in this study are higher than the actual vapor pressure in the chamber because of the flow conditions. An exact way is to measure the partial pressure of the solvent with mass spectrometry at the chamber outlet, which was utilized by Shelton et al [87].

**Figure 2:**
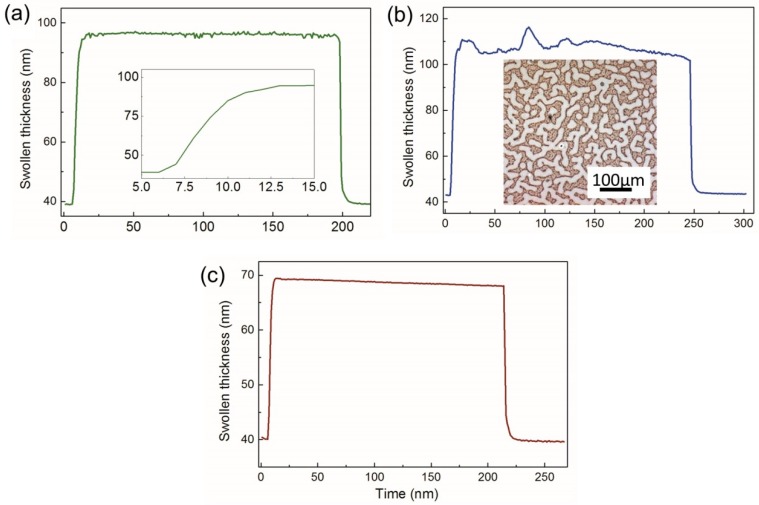
(**a**–**c**) Examples of the swelling curves during continuous annealing process. Thickness of swollen films was monitored by in situ ellipsometry. Inset in (**a**) illustrates the initial fast regime of the solvent uptake. Inset in (**b**) is an optical image of Polystyrene-*b*-poly(2-vinyl pyridine) (PS-PVP) film showing characteristic terracing upon condensation of the solvent on the film surface in the course of annealing.

A part of the stainless steel tubing is immersed in the thermostat bath Lauda E 100 (Lauda Dr. Wobser GmbH&CO.KG, Lauda-Königshofen, Germany) for controlling the temperature of the vapor (*T*_v_) (blue-marked area in Figure 1a), and a part of the tubing is exposed to the laboratory atmosphere, which presents a potential drawback of the system due to possible heat transport between thermostated vapor and the environment. Channel 3 is designed for the studies of the annealing in the mixed solvent vapors [26]. The second thermostat (Lauda RE 204) controls the temperature of the substrate with the polymer film on top (*T*_s_). The quenching of the films is achieved by closing Chanel 2 and purging dry nitrogen through the chamber, so that the film thickness drops down more than 95% to its initial value in a dry state within less than a minute (Figure 2). The stability of the annealing system in this dynamic set-up is provided by a constant flow through the chamber, which can compensate the possible leakage of the system. Moreover, such a set-up has the additional possibility to create overpressure in the chamber, i.e., to saturate the film with the solvent beyond the equilibrium adsorption level at normal conditions, thus creating new morphological structures [72].

Two annealing chambers were used in the studies, one from stainless steel and one from Teflon (PTFE). Their descriptions are presented in Figure 1b,c. For the monitoring of the swollen film thickness with spectroscopic ellipsometry, the chambers are equipped with optical windows to allow the incident and the reflected light beam at an angle of 70°. Both chambers have inner heating tubing at the bottom connected to the thermostat to maintain the temperature of the substrate *T*_s_ independently from that of the vapor *T*_v_. To avoid possible condensation of the solvent on the film surface, the temperature of the substrate should be maintained at least 1 °C higher than the vapor. The thermal conductivity of PTFE is ~0.245 W/m·K, and that of stainless steel is ~14–15 W/m·K. Fast thermal conductivity of the material may increase the sensitivity of the inner environment to the temperature outside the chamber. Apart from the materials, the chambers differ in the total volume and in the position of the inlets and outlets for the tubing. The volumes of steel and Teflon chambers are ~60 mL and 1.2 mL, respectively.

### 2.5. In-Situ Monitoring of the Film Thickness

In order to gain a deeper understanding of the process of the solvent uptake by thin polymer films, it is indispensable to have precise control over the procedure parameters, such as the temperature of the substrate and of the solvent vapor, the values of the total and the partial vapor pressures, and film thickness during swelling. Even dynamic characteristics of the annealing procedure such as the total gas flow through the chamber, the volume and the geometry of the chamber, the rate of the build-up pressure, and the velocity of the final quench all crucially affect the swelling and hence the efficiency and the reproducibility of the annealing procedure. 

A crucial condition to have control over the swelling process is to monitor the development of the swollen thickness in time. This is typically done using ellipsometry [34,71], as in the set-up reported here, or optical reflectometry measurements [30]. Additional valuable information on the microphase separated structures of block copolymers and their dimensions in thin films is gained when real-time grazing incidence small angle X-ray scattering (GISAXS) is utilized in combination with controlled solvent annealing [18,88]. Figure 2 presents several examples of captured swelling curves, each containing important information about the process/system stability. A typical swelling curve of thin film of a linear coil polymer consists of a fast build-up stage (inset in Figure 2a) and a steady-state swelling, which should be maintained on a constant level during the whole annealing process. We note that the swelling curves of glassy networks look principally different, showing a slow continuous growth of the solvent uptake [71]. Also, the deswelling of the films of linear polymers upon quench proceeds fast and with almost no hysteresis (Figure 2a). Irregularities in the measured swollen film thickness such as strong fluctuation (Figure 2b) or constant decrease of the swelling thickness in the course of annealing (Figure 2c) require specific attention to the particular measurement. For example, thickness instability can be a result of condensation of the solvent on the film surface. The apparently uncontrolled jumps in the swelling are probably related (in this case) to unreliable fitting of the ellipsometric data. In block copolymer films, the results of such an artifact can be seen as a non-typical pattern of the formed terraces on the film surface (inset in Figure 2b) [34]. The reasons for the type of swelling behavior as in Figure 2c can be attributed to a decreasing level of the solvent in the vial (see Figure 1a), to changes in the temperature of the environment upon long term processing, or to the leakage in the system.

The time-period and conditions for effective annealing of homopolymer films are not feasible to evaluate. In the case of wrongly chosen parameters, rupture of the film due to dewetting may occur. Dewetting is a dynamic process that completes the dynamics of chain equilibration and is significantly accelerated in the presence of a solvent in the film. In contrast to the films of homopolymers, the visualization of the evolved translational and morphological order of block copolymer microdomains allows for deriving direct conclusions on the efficiency of the annealing, i.e., on the closeness of the system to the thermodynamic equilibrium as well as on the chain conformations [48,66]. At the same time, spontaneous roughening of the free surface (macroscopic terrace formation) of multilayer-thick block copolymer films can be followed in time as a measure of the chain dynamics [41,66,89] or even as a measure of the interfacial interactions with the substrate [90,91].

## 3. Results and Discussion

### 3.1. Choice of the Solvent and of the Swelling Conditions

The physical properties of the employed solvent have to be taken into account when deciding on the annealing conditions. Temperature dependence of the saturated pressures of toluene and chloroform (shown in Figure S1, Supporting Information) suggests that, at 25 °C, the saturated pressure of chloroform is about seven times higher than that of toluene (26.22 and 3.79 kPa, respectively).

**Figure 3:**
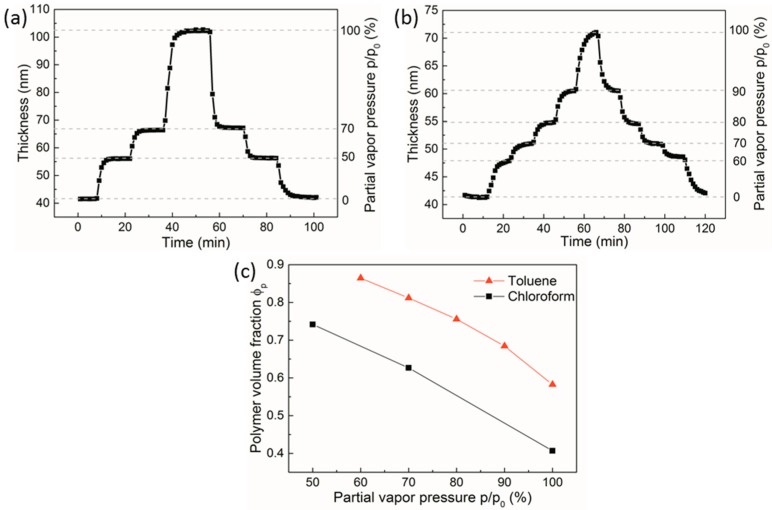
Swelling kinetics of PS-PVP ~40 nm thick films in (**a**) chloroform and (**b**) toluene vapor under stepwise increase of the partial vapor pressure by mixing the flows of Channel 1 and Channel 2 (Figure 1). *T*_v_ and *T*_s_ were kept constant at 19 and 20 °C, respectively. (**c**) Polymer volume fraction of PS-PVP films in chloroform (black square) and in toluene (red triangle) versus partial vapor pressure of the respective solvent.

Figure 3 presents swelling kinetics of PS-PVP films in chloroform and toluene vapors upon stepwise increase of the partial vapor pressure at constant temperatures *T*_v_ and *T*_s_. In chloroform vapor (Figure 3a), the film achieves an equilibrium thickness and steady-state swelling within ~10 min at each partial vapor pressure, which is faster than in toluene vapors (Figure 3b). Figure 3c summarizes the swelling data at saturation for PS-PVP films presented in Figure 3a,b. The solvent fraction (1 − Φ_p_) in the swollen films at 70% p/p_0_ is two times higher for chloroform (37% for chloroform versus 19% for toluene), which reflects both lower toluene vapor pressure and its poor solvent quality toward P2VP block. We note that quantitative evaluations of the solvent concentration in block copolymer films and the related degree of segregation are extremely important for the efficiency of the annealing process. On one hand, low solvent concentration requires longer annealing times. On the other hand, in the case of a high degree of swelling and a low molecular weight of a block copolymer, the condition above order-disorder transition (ODT) could be achieved, thus the removal of the solvent would result in a dynamic quench from a disordered state. In this case, the microphase separated pattern is represented as disordered structures, irrespective of the duration of the annealing [34]. Such directional quench has been shown to be a promising route to induce vertical orientation of microdomains [49,92,93]; however, the procedure requires deeper understanding and optimization with regards to the reproducibility and defects annihilation. In this respect, equilibration of structures under controlled solvent uptake, which provides intermediate or strong segregation regimes, offers a number of advantages in fabrication of ordered patterns, such as low line edge roughness of the resulting structures [75,94], fine control over non-bulk morphologies [95,96], and minimization of the risks of dewetting [66,97].

### 3.2. Effect of the Temperature Variation on the Swelling Behavior of Polymer Films

There are two principle methods to adjust the concentration of the solvent vapors in the chamber: (a) by mixing the flows from Channels 1 and 2 under constant temperature of the substrate *T*_s_ and the vapor *T*_v_, and (b) by tuning the temperature of the vapor/substrate at constant flows, which is denoted as solvo-thermal annealing. While the first approach is more robust with regards to possible fluctuations of the environmental temperature and creates a permanent flow within the chamber, the second approach is promising in view of a stronger influence on the segmental chain dynamics, as is described below. In the case of the temperature control, the pressure of the solvent vapor in the vessels can be calculated according to the Clausius-Clapeyron equation:

ln(p/p_0_) = −Δ*H*_vap_/R·(1/*T* − 1/*T*_0_) (1)

where R is the ideal gas constant, Δ*H*_vap_ is the evaporation enthalpy, and p_0_ and p indicate the saturated pressure of the solvent at temperatures *T*_0_ and *T*, correspondingly. Therefore, the pressure of solvent at a given experimental condition (*T*_s_ and *T*_v_) can be calculated.

**Figure 4:**
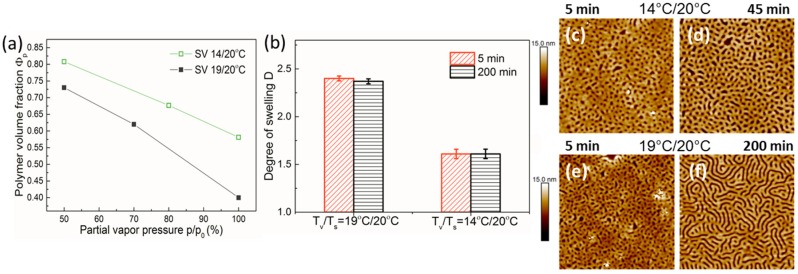
(**a**) Polymer volume fraction Φ_p_ of PS-PVP films (indicated as SV) with a dry thickness of ~40 nm upon stepwise increase of p/p_0_ of chloroform at indicated temperature sets *T*_v_/*T*_s_. Φ_p_ is calculated from the measurements of the dry and the swollen film thicknesses as h_dry_/h_sw_. (**b**) Degree of swelling D of 40 nm thick PS-PVP films annealed at 100 sccm flow of 100% p/p_0_ of chloroform after 5 min and after 200 min of processing under *T*_s_ = 20 °C and *T*_v_ = 14 or 19 °C, as indicated. Scanning force microscopy (SFM) height images of the surface morphology in PS-PVP films after 5 min (**c**,**e**), 45 min (**d**), and after 200 min (**f**) of annealing at indicated temperatures. The scan area is 3 × 3 µm^2^.

To demonstrate the importance of control over the temperature of the vapor, we present the results of the swelling of thin block copolymer films obtained at a constant temperature of the substrate of *T*_s_ = 20 °C and a temperature of vapors *T*_v_ of 14 and of 19 °C (Figure 4). In both cases, the solvent concentration in the vapors was additionally controlled by mixing the flows of Channel 1 and Channel 2 (Figure 1). These results clearly indicate that, without environmental control over the solvent vapor, the reproducibility and the stability of the annealing conditions suffer from temperature fluctuations. The higher solvent content in PS-PVP films at *T*_v_ =19 °C can be expectedly attributed to the strong temperature dependence of the chloroform vapor pressure (Figure S1). The artifacts caused by temperature fluctuations are more pronounced for solvents with strong temperature dependence of the vapor pressure, such as chloroform [34].

The reproducibility of the swelling conditions under varied temperature of the vapor is assessed in Figure 4b. Displayed is the averaged degree of swelling D (*h*_sw_/*h*_dry_, i.e., the reverse of Φ_p_) for 40 nm PS-PVP films after short time processing (5 min) and annealing for 200 min. We emphasize two observations. First, the degree of swelling, which built up in 5 min, was very close to that after the end of the processing, confirming the high degree of stability of the environmental conditions. Second, the swelling experiments were repeated within several months and showed high reproducibility. At the same time, a slightly larger error bar for the *T*_v_14 °C/*T*_s_20 °C set indicated that environmental conditions at *T*_v_ =14 °C, which were far from the room temperature (RT), were more difficult to reproduce, since even a slight deviation in the RT had a large effect on the thermal transfer between the tubing system and the media. The reproducibility issue in an annealing device with a different type of temperature-regulated vapor pressure was assessed by Ogieglo et al. [7]. SFM images in Figure 4 illustrate the development of the morphology of high molecular weight block copolymer PS-PVP during annealing and serve to evaluate the progress of the equilibration process as well as chain dynamics in the swollen films. In both cases, the initial spherical morphology transferred within the first 5 min into dot-like patterns. Longer annealing led to the development of mesh-like (Figure 4d) and striped patterns (Figure 4f). An improved translational order of the microdomains in the latter case was attributed to a larger swollen film thickness rather than to a reduced viscosity of the solution. At this point, we described the developed pattern as an “indicator” of the chain dynamics in the processed films. A more detailed structure interpretation is discussed below in Figure 6. Effects of the annealing chamber on the solvent uptake are presented in Figure S2 (Supporting Information).

**Figure 5:**
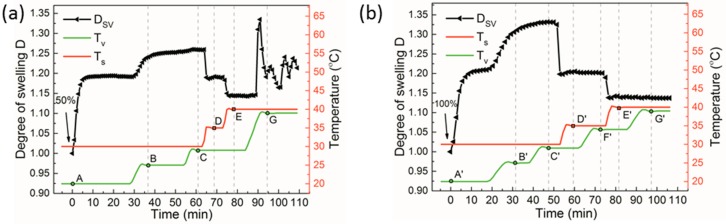
Swelling kinetics (left hand axis: black curves) of ~40 nm thick PS-PVP films (**a**) under chloroform vapor with partial vapor pressure of 50%, and (**b**) under toluene vapor with p/p_0_ of 100% at stepwise increasing of *T*_v_ and *T*_s_ (right hand axis: green curves and red curves, correspondingly). Temperature set *T*_v_/*T*_s_ are the following: (A,A’)—20 °C/30 °C, (B,B’) —25 °C/30 °C, (C,C’) —29 °C/30 °C, (D,D’) —29 °C/35 °C, E—29 °C/40 °C, E’—34 °C/40 °C, F’—34 °C/35 °C, (G,G’) —39 °C/40 °C.

We further studied the approach of independent variations of *T*_v_ and *T*_s_ with regards to the response of the system to the applied conditions. Figure 5 presents the evolution of the degree of swelling of ~40 nm thick PS-PVP films upon changes of *T*_v_ or *T*_s_. The measurements were performed under 50% p/p_0_ of chloroform (Figure 5a) and under 100% p/p_0_ of toluene (Figure 5b) in order to compensate the intrinsic differences in the temperature dependence of the respective solvents (Figure S1, Supporting Information). In both experiments, the following scheme was used: at point A, A’, the system was set to temperature conditions *T*_v_/*T*_s_ of 20 °C/30 °C. In the following steps B, B’ and C, C’, the temperatures of the vapor were increased to 25 and 29 °C, respectively. The temperature data were read from the data panel of the respective thermostat. At point D, D’, the temperature of the substrate was raised to 35 °C and then to 40 °C at point E, E’. For the experiment in toluene vapor, the temperature of the vapor was increased further to 34 °C at point F’ (Figure 5b), while it kept constant at 29 °C in the experiment in chloroform vapor (Figure 5a). In the final step G, G’, the temperature of the vapor was increased to 39 °C. These experiments had two purposes: (i) to evaluate the rate of response of the instrumentation to the change of the temperature conditions (read from thermostats), and (ii) to compare the rate of physical change of the system, i.e., the dynamics of the sample response, to the changed environmental conditions. As seen in Figure 5, the changes in the temperature and in the respective response of the polymer film toward changed environmental conditions were quite synchronic, confirming high sensitivity of the solvent uptake to the temperature conditions, except for the highest studied temperature range. Below is a detailed discussion of the swelling behavior.

At point A (Figure 5a), *T*_s_ and *T*_v_ were set to 30 and 20 °C, respectively. The equilibrium swelling at 50% p/p_0_ of chloroform was achieved within 10 min and remained stable until (at point B) *T*_v_ was increased to 25 °C. The swelling built up first in a two-step mode: a fast stage when ~98% of the equilibrium degree of swelling was achieved and a slow mode when the steady-state swelling was not yet achieved after 25 min. The slow continuous increase in the swollen thickness could be associated with the retarded build-up of the vapor pressure in the chamber as well as with the structural rearrangements in the block copolymer film [46]. At point C, the temperature of the vapor was further increased up to 29 °C, still being 1 °C lower than that of the substrate. However, no clear response of the polymer film could be measured. We believe that *T*_s_ was high enough to cause condensation of the vapor inside the tubes, which were exposed to the colder laboratory environment. This artifact may have resulted in effectively slow increase of the vapor pressure in the chamber. At point D, the temperature of the substrate T_s_ was increased to 35 °C. This environmental change was expectedly accompanied by the drop in the degree of swelling from 1.25 to 1.18. At point E, the substrate temperature *T*_s_ was further increased up to 40 °C with T_v_ being constant, and the film responded synchronically with the environmental changes (*T*_s_) again by decrease in the solvent uptake. At point G, *T*_v_ was increased up to 39 °C, which was much higher than room temperature, thus the system was immediately destabilized due to the condensation of the solvent in the tubing and at the film surface. These conditions defined an upper temperature set for a given set-up and solvent to perform thermally controlled annealing.

The choice of the solvo-thermal annealing conditions depends on the nature of the solvent. Along with chloroform, toluene is widely used in annealing processes, especially in a mixture with aliphatic solvents to equilibrate styrenic block copolymers with high χ parameter, such as polystyrene-*b*-poly dimethylsiloxane [29,36]. The swelling behavior in toluene vapors (Figure 5b) upon stepwise increase of *T*_s_ (A’, B’, C’) was qualitatively similar to that in the vapors of chloroform, including the absence of the response of the film to the increase of the environmental temperature up to 29 °C (point C’). Also, increase of *T*_s_ (D’, E’) caused a fast drop down of the degree of swelling from 1.33 to 1.20 and then from 1.20 to 1.14. When T_v_ was increased at points F’ and G’, the polymer film did not respond to this environmental change, although the whole set-up seemed to be more resistant to the condensation of the vapor in the tubing of the system and on the sample. Further improvement of the experimental set-up for the controlled solvo-thermal annealing requires better isolation of the system parts from the laboratory environment. 

We note that the studies of swelling behavior under elevated substrate temperature are directly relevant to the development of warm solvent vapor annealing, which has advantages in film processing that were already qualitatively reported [30,37,75]. The potential of this method is envisaged in enhanced segmental mobility of the polymer chains at an elevated temperature. This effect should compensate the decrease in viscosity as a result of the reduced solvent uptake. However, changes of the polymer-polymer and the polymer-solvent interaction parameters for a particular system upon varying solvent concentration also have to be taken into account. Our study suggests a methodological approach to quantitatively assess solvent uptake and chain dynamics at elevated temperature conditions. Importantly, this approach is applicable to semi-crystalline conjugated polymers as well as to complex composite materials under confinement.

**Figure 6:**
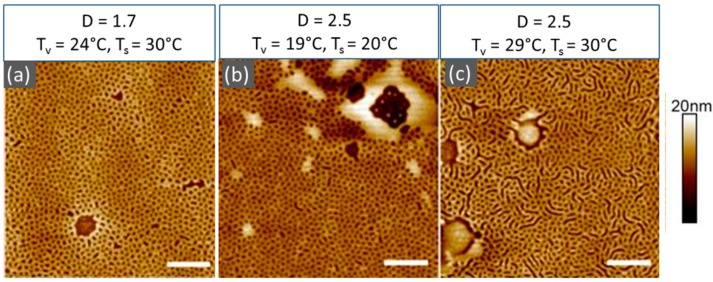
SFM height images of the surface morphology of 40 nm thick PS-PVP films processed under indicated temperature sets *T*_v_/*T*_s_. D is the degree of swelling calculated from the measurements of the swollen and the dry film thicknesses as *h*_sw_/*h*_dry_. The swelling curves for the films shown in (**a**), (**b**), and (**c**) are presented in Figure 2 (c), (a), and (b), respectively.

Shown in Figure 6 are the height SFM images of the surface structures, which developed in 40 nm thick PS-PVP films upon annealing at varied temperature set *T*_v_/*T*_s_, as indicated. We note that the studied film thickness was significantly below the lamella period of this block copolymer in bulk (~117 nm). This resulted in significant confinement and frustration of the morphological structures in swollen films as well as considerably retarded chain dynamics (defect annihilation) during processing. A more detailed analysis and identification of the frustrated morphologies will be presented elsewhere [98]. Here, we focus on the effect of the temperature of the substrate on the annealing process. Comparison of the images in Figure 6a,c, i.e., at the substrate temperature *T*_v_ = 30 °C and varied temperature of the vapors, indicated the same trend as at *T*_v_ = 20°C (Figure 4d,f)—a transition from a mesh-like pattern at a lower degree of swelling (higher polymer volume fraction) to a stripe pattern with an increased degree of swelling. Therefore, the effect of elevated substrate temperature was not feasible to elaborate at these conditions. 

Figure 6b,c compares surface structures in PS-PVP films processed at the same degree of swelling (~2.55) and different substrate temperatures. At *T*_s_ = 20 °C, a mesh-like pattern was developed, while at 10 °C higher temperature, the structures during a similar duration of annealing started to evolve into a striped pattern, which could be attributed to the formation of more energetically favorable up-standing lamella [98]. Therefore, these results confirm the potential of solvo-thermal annealing and offer a tool to optimize the processing of high molecular weight and semi-crystalline polymers as well as high χ-parameter block copolymers.

### 3.3. Evaluation of the Polymer-Solvent Interaction Parameter

**Figure 7:**
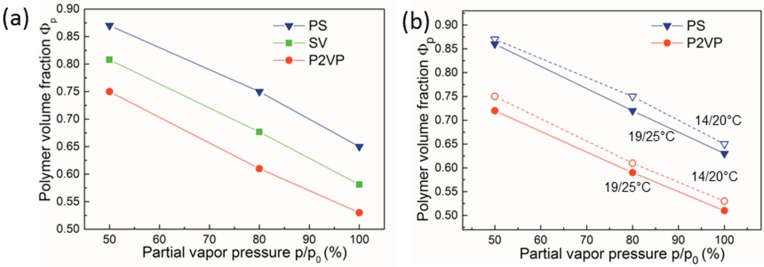
Polymer volume fraction Φ_p_ of PS-PVP (SV), PS, and poly(2-vinyl pyridine) (P2VP) films with a dry thickness of ~40 nm upon stepwise increase of p/p_0_ of chloroform: (**a**) in swollen PS-PVP (green squares), PS (blue triangles), and P2VP (red dots) films all at a temperature set *T*_v_/*T*_s_ of 14 °C/20 °C; (**b**) in swollen PS (blue) and P2VP (red) films at *T*_v_/*T*_s_ = 14 °C/20 °C (empty symbols, dashed line) and at *T*_v_/*T*_s_ = 19 °C/25 °C (solid symbols, solid lines). Φ_p_ is calculated from the measurements of the dry and the swollen film thicknesses as *h*_dry_/*h*_sw_.

When swelling experiments are performed in a systematic and reproducible manner, they provide valuable quantitative information regarding solvent-polymer interactions, i.e., the selectivity of the solvent toward block copolymer components. Figure 7a presents systematic measurements of the swollen films of homopolymers PS and P2VP and of block copolymer PS-PVP, all at similar annealing conditions as a function of the partial vapor pressure of chloroform. As can be seen from the data, chloroform had a clear selectivity to the P2VP block, since P2VP film adsorbed up to 15% more solvent than the PS film. Furthermore, as seen in Figure 7b, the solvent uptake by PS-PVP block copolymer film could be considered as an average of that by PS and P2VP phases, taking into account a symmetric volume composition of the PS-PVP diblock copolymer. We note that, in earlier studies, chloroform was considered as an almost neutral solvent to the components of the PS-PVP block copolymer [73].

We further assessed the swelling behavior of the homopolymer films under two sets of temperatures *T*_v_/*T*_s_ (Figure 7b). For all studied conditions, the values of polymer volume fraction Φ_p_ at the temperature set *T*_v_/*T*_s_ = 19 °C/25 °C were systematically smaller than those at the lower temperature of the vapors (*T*_v_/*T*_s_ = 14 °C/20 °C). This observation suggests that the increase in the solvent uptake as a result of the increased vapor pressure dominated the reduction in solvent sorption by the heated sample (Figure 5). We note that the selectivity of the chloroform to P2VP block was preserved at both annealing conditions.

Equation (2) allows for the evaluation of polymer-solvent interaction parameters in polymer films based on the measurements of Φ_p_ at each swelling condition, as was earlier reported by several groups [41,57,60]:

ln(P/P_0_) = χ_P,S_ Φ_p_^2^ + ln(1 − Φ_p_) + (1 − 1/N) Φ_p_, (2)

where N is the total degree of polymerization and χ_P, S_ is the Flory-Huggins interaction parameter between the polymer and the solvent; P/P_0_ is the normalized partial vapor pressure, P is the partial vapor pressure of the solvent in the chamber, and P_0_ is the saturated partial pressure of the corresponding pure solvent.

**Figure 8:**
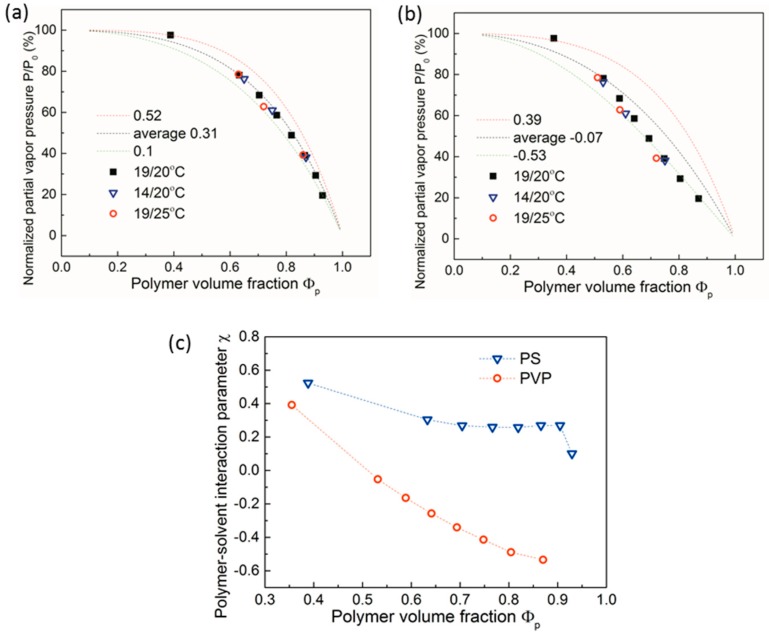
Polymer volume fraction Φ_p_ in swollen (**a**) PS and (**b**) P2VP films with an initial thickness of ~40 nm versus normalized partial vapor pressure P/P_0_ of chloroform at indicated temperatures. Data points were measured at *T*_v_/*T*_s_ of 19 °C/20 °C (black squares), 14 °C/20 °C (blue hollow triangles), and 19 °C/25 °C (red hollow circles), respectively. Dashed curves are calculated fits according to Equation (2) with a fixed polymer–solvent interaction parameter χ (**a**) 0.13 (green), 0.68 (red), and 0.41 (black), and (**b**) −0.51 (green), 0.39 (red), and 0.02 (black). (**c**) Polymer–solvent interaction parameter c as a function of polymer volume fraction Φ_p_ of PS (blue hollow triangles) and P2VP (red hollow circles) ~40 nm thick films in chloroform vapor with step wise increasing partial vapor pressure.

Figure 8a,b presents experimental data points of the polymer volume fraction in swollen PS and P2VP films versus partial vapor pressure P/P_0_ of the chloroform under different temperature sets. The dashed lines represent fits according to Equation (2) keeping χ constant at three selected values. The partial vapor pressure was normalized with regards to the temperature, as described in Supporting Information. The range of the solvent-polymer interaction parameter χ_P,S_ as a function of the dilution of the polymer (i.e., versus P/P_0_) was calculated using Equation (2) and is presented in Figure 8c. For PS films, the values of χ varied from 0.52 to 0.1 with increasing polymer concentration in the range of Φ_p_ 0.4–0.9. These values were in a good agreement with the literature data [99]. PVP films showed similar evaluation results in the range of χ from −0.53 to 0.39. While lower values of χ confirmed a better quality of chloroform to the PVP phase as compared to PS phase, the negative values of χ indicated strong polar attractive interactions between the PVP phase and chloroform. 

Each curve in Figure 8a,b was calculated using fixed χ values of the lower and the upper borders of the evaluated range as well as the middle value. The data points for PS in Figure 8a were quite compact, showing no measurable dependence on the temperature conditions within the studied range, and they could be well fitted with an averaged value of χ ~ 0.31, except for highly swollen films. Obviously, our approach of measuring the overall solvent uptake does not provide all the information necessary to gain an improved understanding of the interplay between kinetic and thermodynamic effects that impact the swelling behavior of polymer chains under confinement. Involving more sophisticated methods such as as neutron scattering would allow detailed insight into possibly uneven solvent distributions through the film and into analyses of the plasticization of PS in thin swollen films [85]. Here, we demonstrate that the highly controlled annealing approach allows retrieval of qualitative differences in the swelling behavior between two studied homopolymers. As seen in Figure 8b, the data points for PVP films looked more process-dependent compared to PS films. At high polymer volume fractions above 0.6, the polymer-solvent interactions seemed to be governed by polar interactions, irrespective of the studied temperature range. With increasing solvent concentration, the type of interaction changed, suggesting that the solvent selectivity towards PVP phase decreased. We note that Equation (2) does not take into account specifics of thin films under confinement, such as differences in the chain conformation in the vicinity of the solid support and the free surface or inhomogeneous solvent distribution in the film as a result of, e.g., accumulation of polar solvent in the vicinity of the polar substrate; therefore, the effect of temperature condition on the swelling behavior of thin films can be weakened on the macroscopic scale.

## 4. Conclusions

Solvent annealing has been used to improve self-assembly of block copolymer films for more than 20 years, since its application was first reported by Thomas and co-workers in 1998 [100]. Achieving high precision and reproducibility of the annealing process is still an experimental challenge. In this study, we demonstrated the effects of environmental and experimental factors on the stability of the processing conditions. Monitoring the swollen film thickness is a crucial condition to gain understanding and control over the solvent vapor annealing.

Process parameters such as total vapor flow, dimension and material of the chamber, solvent property, and temperature were assessed and discussed. We demonstrated that the temperature of the substrate and the temperature of the vapor are the key factors to controlling the solvent uptake and the equilibration dynamics in polymer films. Swelling behaviors of homopolymer and block copolymer films were systematically analyzed to evaluate the solvent selectivity to the block copolymer components as an example of one of the multiple scientific usages of the method of controlled solvent vapor annealing. Temperature-controlled swelling of polymer films (solvo-thermal annealing) was introduced on a quantitative basis. The methodological approach reported herein is applicable to semi-crystalline conjugated polymers as well as to complex composite materials under confinement.

## Supplementary Materials

The following are available online at https://www.mdpi.com/2073-4360/11/8/1312/s1, Figure S1: Saturated pressure of chloroform and toluene at various temperatures under 1 atm, Figure S2: Effect of the annealing chamber on the solvent up take.
