# Supplementary material for: Temperature-Controlled Solvent Vapor Annealing of Thin Block Copolymer Films
Source: Polymers (Basel). 2019 Aug 6;11(8):1312. doi: 10.3390/polym11081312 (PMC6722758; doi:10.3390/polym11081312)
Supplement: Supplementary file 1 [file polymers-11-01312-s001.pdf]

polymers

Temperature-Controlled Solvent Vapor Annealing of Thin Block Copolymer Films

Xiao Cheng [1,2], Alexander Böker [1,2] and Larisa Tsarkova [3,4,*]

1 Fraunhofer Institute for Applied Polymer Research IAP, Geiselbergstr. 69, 14476 Potsdam-Golm, Germany
2 Lehrstuhl für Polymermaterialien und Polymertechnologie, University of Potsdam, 14476 Potsdam-Golm, Germany
3 Deutsches Textilforschungszentrum Nord-West (DNTW), Adlerstr. 1, 47798 Krefeld, Germany
4 Chair of Colloid Chemistry, Department of Chemistry, Moscow State University, Leninskie Gory 1-3, 119991 Moscow, Russia
* Correspondence: tsarkova@colloid.chem.msu.ru or tsarkova@dtnw.de; Tel.: +49-02151-843-2016

Supporting Information

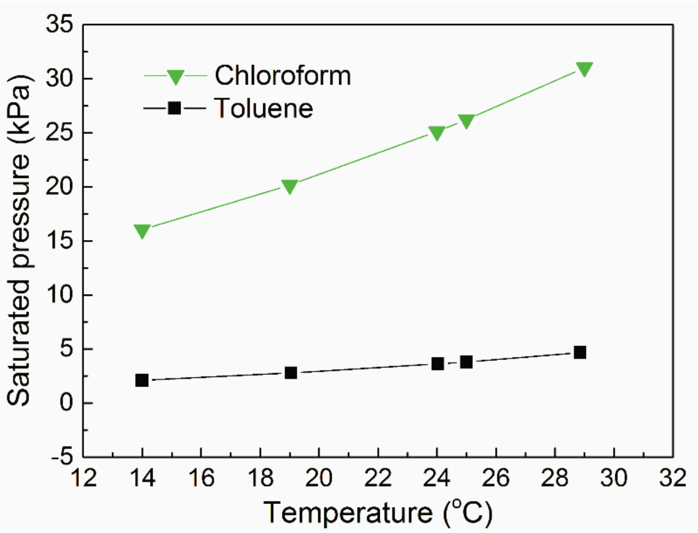

Figure S1. Saturated pressure of chloroform and toluene at various temperatures under 1 atm [1,2].

Effect of the annealing chamber

The swelling experiments on ~40 nm-thick P2VP films have been performed at absolutely identical conditions using the same tubing and thermostating set-up but different annealing chambers. Figure S2 presents polymer volume fraction Φ_p in swollen films as a function of p/p_0 of chloroform. As can be seen, Φ_p in the steel chamber is systematically lower than that in Teflon chamber, with the difference in the swollen thickness at 100% p/p_0 as large as ~10 nm.

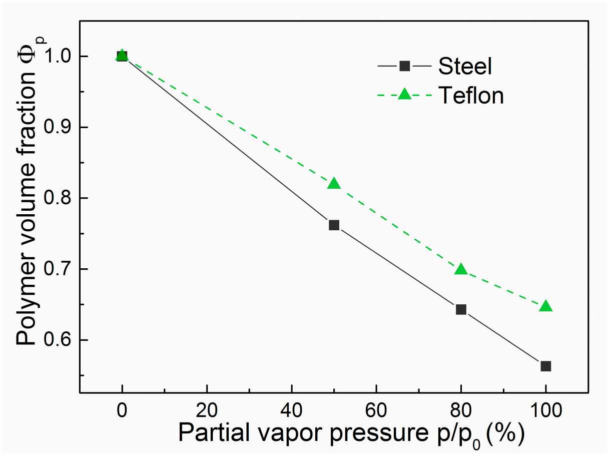

Figure S2. Polymer volume fraction of ~40 nm thick P2VP films as a function of partial vapor pressure of chloroform. The annealing was performed under identical conditions at 100 sccm total flow, at T_v/T_s 14°C/20°C temperature range in steel (black squares) and Teflon (green triangles) chambers.

As presented in the experimental section, the material of the chamber, the inner volume and the positions of the input and output outlets for the tubing are three main differences of the steel and Teflon chambers. The huge difference in the thermal conductivity of the material can lead to opposing effects with regards to the possible influences of the temperature variation. It is reasonable to suppose that the enhanced heat exchange in the steel chamber will lead to effective increase of both set temperatures 14°C/20°C. While increase of T_v results in a higher degree of swelling, the increase of the substrate/polymer temperature has an opposite influence on the solvent uptake (Figure 5). The extent of both effects is not feasible to evaluate without additional thermo-censing devices. Further, the larger volume of the steel chamber may lead to a retarded kinetics to achieve steady-state regime, but we did not observe this effect within the time resolution of our measurements. Furthermore, the differences in the inner volume geometry, as well as in the position of flow tubes may define the type and velocity of the flow through the chamber. The Teflon chamber has a volume of ~1.2 mL with input and output tubes on the opposite sides of the chamber, so that the solvent vapor is supposed to have laminar type of flow with enhanced velocity. In contrast, we assume that the inner volume, as well as the positions of inlet and outlet openings in the steel chamber provide conditions for turbulent flow with decreased velocity. Since input/output tubes are not thermo-isolated, the vapor pressure in the steel chamber is supposed to be higher than that in the Teflon chamber. The outcome of the above tests is that annealing experiments performed under controlled conditions using the same set-up and the same laboratory environment show high levels of reproducibility. However, even slight (uncontrolled) changes in system parts or in the environment affect the absolute values of the solvent concentration in the film, so that the

experimental procedures and results developed by different groups are not directly convertible. In laboratory practice, the influence of all these factors on the accuracy of the experiments in most cases is not properly considered.

References

[1] Ogieglo, W., Stenbock-Fermor, A., Juraschek, T. M., Bogdanova, Y., Benes, N., Tsarkova, L. A. Synergic Swelling of Interactive Network Support and Block Copolymer Films during Solvent Vapor Annealing. *Langmuir* **2018**, *34*, 9950–9960.
[2] Ian M. Smallwood. *Handbook of Organic Solvent Properties*; Elsevier, 1996.
[3] Emerson, J. A., Toolan, D. T. W., Howse, J. R., Furst, E. M., Epps, T. H. Determination of Solvent–Polymer and Polymer–Polymer Flory–Huggins Interaction Parameters for Poly(3-hexylthiophene) via Solvent Vapor Swelling. *Macromolecules* **2013**, *46*, 6533–6540.
[4] R.OA. Orwoll, P.A. Arnold. Polymer-Solvent Interaction Parameter. Chapter 14 in Mark, J. E., Physical Properties of Polymers Handbook. AIP Press: New York, 1996.

© 2019 by the authors. Licensee MDPI, Basel, Switzerland. This article is an open access article distributed under the terms and conditions of the Creative Commons Attribution (CC BY) license (http://creativecommons.org/licenses/by/4.0/).
